# Ultrasonographic examination of masticatory muscles in patients with TMJ arthralgia and headache attributed to temporomandibular disorders

**DOI:** 10.1038/s41598-024-59316-9

**Published:** 2024-04-18

**Authors:** Yeon-Hee Lee, Hyungkyu Bae, Yang-Hyun Chun, Jung-Woo Lee, Hee-Jin Kim

**Affiliations:** 1 Department of Orofacial Pain and Oral Medicine, Kyung Hee University, Kyung Hee University Dental Hospital, #613 Hoegi-dong, Dongdaemun-gu, Seoul, 02447 South Korea; 2 Division in Anatomy and Developmental Biology, Department of Oral Biology, Human Identification Research Institute, BK21 FOUR Project, Yonsei University College of Dentistry, Seoul, South Korea; 3 Department of Oral and Maxillofacial Surgery, School of Dentistry, Kyung Hee University, Seoul, 02447 South Korea

**Keywords:** Ultrasonography, Arthralgia, Headache attributed to temporomandibular disorders, Masseter muscle, Temporalis muscle, Temporomandibular disorder, Medical research, Signs and symptoms

## Abstract

This study used ultrasonography to compare the thickness and cross-sectional area of the masticatory muscles in patients with temporomandibular joint arthralgia and investigated the differences according to sex and the co-occurrence of headache attributed to temporomandibular disorders (HATMD). The observational study comprised 100 consecutive patients with TMJ arthralgia (71 females and 29 males; mean age, 40.01 ± 17.67 years) divided into two groups: Group 1, including 86 patients with arthralgia alone (60 females; 41.15 ± 17.65 years); and Group 2, including 14 patients with concurrent arthralgia and HATMD (11 females; 33.00 ± 16.72 years). The diagnosis of TMJ arthralgia was based on the diagnostic criteria for temporomandibular disorders. The parameters of the masticatory muscles examined by ultrasonography were subjected to statistical analysis. The pain area (2.23 ± 1.75 vs. 5.79 ± 2.39, *p*-value = 0.002) and visual analog scale (VAS) score (3.41 ± 1.82 vs. 5.57 ± 12.14, *p*-value = 0.002) were significantly higher in Group 2 than in Group 1. Muscle thickness (12.58 ± 4.24 mm) and cross-sectional area (4.46 ± 2.57 cm^2^) were larger in the masseter muscle than in the other three masticatory muscles (*p*-value < 0.001). When examining sex-based differences, the thickness and area of the masseter and lower temporalis muscles were significantly larger in males (all *p*-value < 0.05). The area of the masseter muscle (4.67 ± 2.69 vs. 3.18 ± 0.92, *p*-value = 0.004) and lower temporalis muscle (3.76 ± 0.95 vs. 3.21 ± 1.02, *p*-value = 0.049) was significantly smaller in Group 2 than in Group 1. An increase in VAS was significantly negatively correlated with the thickness of the masseter (r =  − 0.268) and lower temporalis (r =  − 0.215), and the cross-sectional area of the masseter (r =  − 0.329) and lower temporalis (r =  − 0.293). The masseter and lower temporalis muscles were significantly thinner in females than in males, and their volumes were smaller in patients with TMJ arthralgia and HATMD than in those with TMJ arthralgia alone. HATMD and decreased masseter and lower temporalis muscle volume were associated with increased pain intensity.

## Introduction

Temporomandibular disorders (TMDs) are common orofacial musculoskeletal disorders characterized by pain and dysfunction of the temporomandibular joints and surrounding structures. In 2014, new evidence-based diagnostic criteria for TMD (DC/TMD), TMD was divided into 12 common TMDs: arthralgia, myalgia, local myalgia, myofascial pain, myofascial pain with referral, four kinds of disc displacement disorders, degenerative joint disease, subluxation, and headache attributed to TMD (HATMD)^1^. In the previous version of the research diagnostic criteria for TMD (RDC/TMD) published in 1992, arthralgia was included in group III, and HATMD was not included in classification^2^. In 2018, the International Classification of Headache Disorders, 3rd edition (ICHD-3), defined HATMD as a headache caused by TMD involving the temporomandibular region. HATMD is induced by jaw motion, such as chewing or bruxism, and reproduced on physical examination by stimulation of the upper temporalis muscles and/or passive movement of the jaw^3^, and may be related to temporomandibular joint (TMJ) arthralgia.

TMD encompasses various etiologies and symptoms involving the TMJ and is characterized by TMJ arthralgia. In a systematic review, the overall prevalence of TMD was approximately 31% in adults and 11% in children/adolescents^4^. TMD occurs predominantly in women and is 2.3 times more likely than in men^5^. The prevalence of primary headache as a TMD symptom is estimated to be 56%^6^. However, the prevalence of HATMD is 1–5.7%, which is lower than that of primary headaches^7^. Typical signs and symptoms of TMD include TMJ sounds, TMD pain, restriction of mouth opening, masticatory dysfunction, headache, and tinnitus, and these symptoms may occur alone or in combination^8^. TMJ arthralgia is characterized by spontaneous pain or pain on movements in the TMJ and pain on palpation of the lateral pole or posterior part of the TMJ on the same side^9^. Persistence of joint arthralgia can lead to pathological changes in functionally related joints and structurally adjacent muscles, leading to changes and/or limitations in anatomical function^10,11^. Clinically, TMJ arthralgia can co-occur with HATMD; however, few previous studies have investigated their relationship or clinical characteristics.

HATMD is a type of secondary headache that is common in patients with TMD. The prevalence of HATMD in patients with TMD ranged from 5.4 to 29.3%^7,12^. According to DC/TMD, HATMD is “a headache in the temple area secondary to pain-related TMD that is affected by jaw movement, function, or parafunction, and replication of this headache occurs with provocation testing of the masticatory system”^1^. The ICHD-3 classification assessed the chronological relationship, whether headache occurred or was discovered after the onset of TMD^3^. Based on the source of TMD pain, painful TMD can be broadly divided into arthrogenous and myogenous TMD^13,14^. The relationship between myogenous TMD and HATMD rather than arthrogenous TMD has been discussed earlier in the academia^15,16^. HATMD is common in TMD patients with myogenous TMD. Approximately 62% of chronic myogenous TMD patients had HATMD of DC/TMD criteria^15^. According to Costa et al., myogenous TMD patients with HATMD showed higher pain sensitivity to pressure in the anterior temporal muscle than those without^17^. However, arthrogenous TMD may precede or co-occur with myogenous TMD, and both TMDs can have extensive overlap with headache. A close relationship between painful TMD and headache exists and can be explained by the shared trigeminal pain pathway and peripheral and central sensitization, together with the neuroanatomical connectivity between the three branches of the trigeminal nerve^18^. The relationship between arthrogenous TMD and headache has been suggested indirectly and requires further investigation^19^. In this study, the occurrence of HATMD in patients with arthrogenous TMD, TMJ arthralgia, and the effect of concurrent HATMD on the masticatory muscles were investigated.

Imaging can assess TMJ arthralgia and HATMD. High-frequency ultrasound is excellent for imaging normal muscles and joints, detecting muscular changes, and diagnosing joint diseases^20^. An examination for TMJ arthralgia involves evaluating TMJ or masticatory muscle pain, stiffness, facial asymmetry, and mouth opening limitation. Recently, a detailed examination of the masticatory muscles by palpation has been added, as muscle tenderness may reveal an active disease^21^. However, the TMJ is one of the most difficult joints for clinical and imaging evaluations and has a complex configuration. TMJ bony structure evaluation is possible through conventional radiography, cone-beam computed tomography (CBCT), and CT; however, muscle evaluation is limited to magnetic resonance imaging (MRI) and ultrasonography^22^. Muscle ultrasound is a convenient technique for noninvasive and real-time visualization of normal and pathological muscle tissues. Using the grayscale analysis technique of ultrasonography, neuromuscular disorders could be detected with a predictive value of 90%^23^. Ultrasonography is an accurate and reliable imaging technique for measuring the thickness and cross-sectional area of masticatory muscles in vivo^24,25^; however, studies examining the four major masticatory muscles in patients with TMD are limited.

We hypothesized that ultrasonography-based changes in the thickness and cross-sectional area of the major masticatory muscles—the masseter, upper temporalis, lower temporalis, and medial pterygoid—could predict pain in patients with TMJ arthralgia. Additionally, pain intensity would be higher in patients with arthralgia-induced concurrent HATMD than in those with arthralgia alone, which could be related to changes in the masticatory muscles. To verify this, the clinical characteristics, HATMD status, and ultrasound masticatory muscle parameters in patients with TMJ arthralgia were compared and analyzed.

## Methods

The research protocol for this study was reviewed to ensure compliance with the principles of the Declaration of Helsinki and was approved by the Institutional Review Board of Kyung Hee University Dental Hospital in Seoul, South Korea (KHD IRB, IRB No-KH-DT22015). Informed consent was obtained from all the participants.

### Participants

This study included 100 consecutive patients with arthrogenous TMD (71 females and 29 males; mean age, 40.01 ± 17.67 years) who visited the Kyung Hee University Dental Hospital between June 2022 and September 2022. The patients were divided into two groups: Group 1, comprising the patients with arthralgia alone and Group 2, comprising the patients with concurrent arthralgia and HATMD. Subject selection was based on a standardized clinical examination. The inclusion criteria were as follows: all subjects underwent a physical examination according to the DC/TMD, had TMJ arthralgia^1^, and were aged 18 years or older. TMJ arthralgia, a representative TMD subgroup, was defined as spontaneous pain or pain on movement in the TMJ and pain on palpation of the lateral pole or posterior attachment of the TMJ on the same side. The exclusion criteria were as follows: (1) systemic inflammatory connective tissue disease; (2) history of facial tumor or surgery; (3) pregnancy; (4) occult neuralgia in the orofacial area; (5) local facial infection; and (6) psychiatric or psychological disorders.

### Study design

#### Clinical evaluation

(1) Characteristics of TMD pain

**Figure 1:**
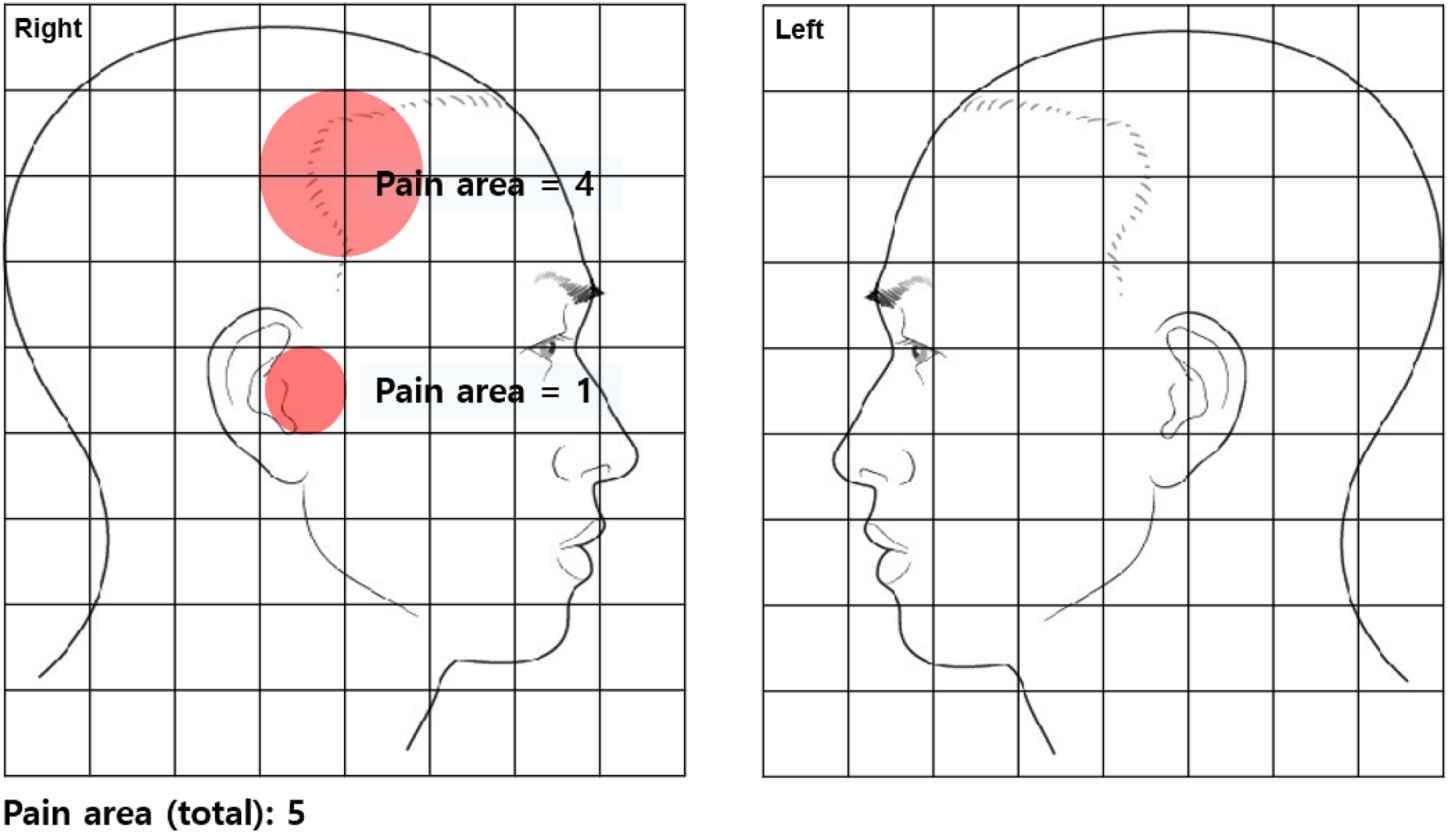
Pain area using overlapping grids on the right and left side of the face. The pain area was calculated by summing the number of grids marked with the area where the patient self-marked their pain.

The duration of pain due to TMJ arthralgia has been reported in days. When the symptom duration was > 6 months (180 days), it was regarded as chronic TMD^26^. TMD pain was scored subjectively by the patients, ranging from 0 (no pain at all) to 10 (the worst pain imaginable) using the visual analog scale (VAS). The pain laterality was classified as right-sided, left-sided, and both sides (bilateral). The pain area was measured using overlapping grids on the right and left facial parts, which was the sum of the grid cells marked by the patient (Fig. 1).

(2) Evaluation of HATMD

HATMD, a TMD subgroup, was diagnosed according to DC/TMD, and the criteria were as follows: (1) history taking: (a) headache in the temple area and (b) headache modified by jaw movement, function, or parafunction; (2) physical examination: (a) confirmation of headache location in the temple area and (b) familiar headache in the temple area with provocation test^1^.

(3) Ultrasonography measurements

**Figure 2:**
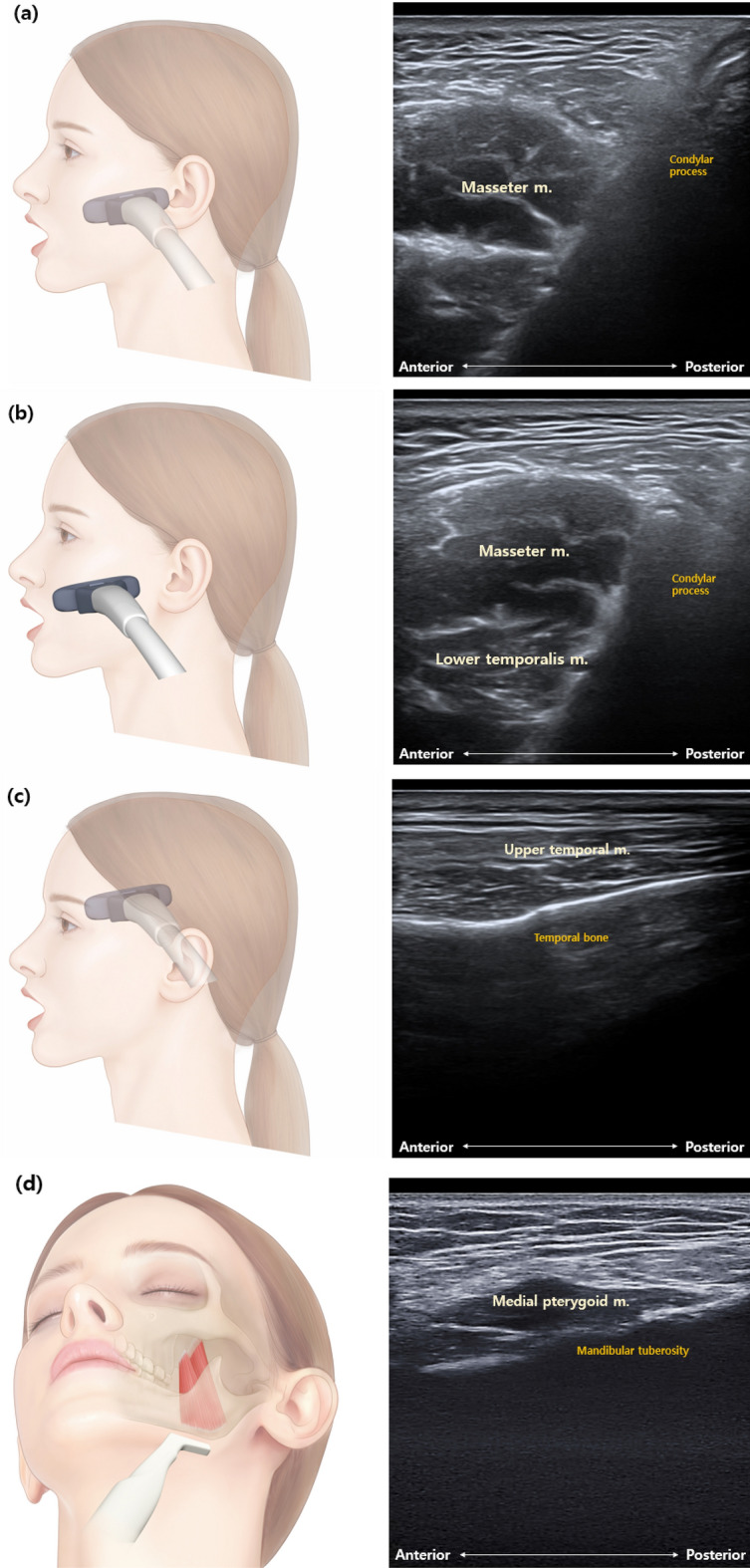
Diagram and actual ultrasound image of each masticatory muscle. (**a**) Masseter, (**b**) lower temporalis, (**c**) upper temporalis, and (**d**) medial pterygoid muscles.

Ultrasonography was performed with a General Electric Versana Active ultrasound system (General Electric, Milwaukee, Wisconsin, USA), using an M12L linear transducer with a pulse frequency of 0–14 MHz. A fixed B-mode setting for grayscale was selected for the musculoskeletal examination. The gain, focus, and depth were individually adjusted per patient. Doppler sensitivity was optimized for low flow with fixed settings (7.5 MHz Doppler frequency, pulse repetition frequency of 0.9 kHz, wall filter of 114 Hz). The ultrasound images of major masticatory muscles were frozen at the following locations (Fig. 2), and the muscle thickness and cross-sectional area of each masticatory muscle on both the right and left sides were measured. Measurements were made using a program built into the Versana Active device. All ultrasound examinations were performed by a young physician investigator (YHL) with relevant training.

(4) Thickness and cross-sectional area of the masticatory muscle

The patients’ masticatory muscles were examined in a relaxed supine position. In the echogenicity of major anatomical structures, normal bone interfaces are hyperechoic, fascia is hyperechoic, and muscles are hypoechoic^27,28^. The masseter muscle’s thickness and cross-sectional area were measured near its middle portion in the inferior zygomatic region. The transducer was placed at the most abundant part of the masseter muscle, parallel to the long axis of the zygomatic arch, between the lower mandibular notch and the inferior border of the mandible. For assessing the lower temporalis muscle, the patient's mouth was opened by 2 cm. The lower temporalis muscle was measured in the optimal position, where a muscle with a clear boundary was found by moving the linear probe from the zygomatic arch toward the mandibular notch. For the upper temporalis muscle, a linear probe was placed parallel to the outer edge of the eyebrow. For the medial pterygoid muscle, after placing the probe parallel to the inferior border of the mandible at the position of the posterior digastric muscle, a slight force was applied from the inside to the outside to measure. All masticatory muscles were investigated extra-orally on both the right and left sides. Muscle thickness was defined as the maximal distance between the outer and inner fasciae and was measured in millimeters. The cross-sectional area of each muscle was measured directly in cm^2^ using the area-measuring tool of the device in the image of the estimated muscle thickness.

(5) Reliability and measurement error

The inter-and intra-observer reliability assessed the degree of agreement between multiple repetitions of a clinical test performed. All parameters on the ultrasound scan were measured twice by two investigators (YHL & YHC), and images were presented randomly to assess the inter- and intra-observer reliability. Inter-class correlation coefficients (ICCs) were calculated, and it was prespecified that correlations between assessments should be > 0.80 for all items. ICCs of 0.83 and 0.90 were estimated in intra-examiner reproducibility measurements. If the ICC did not reach this standard value, it was classified as an additional investigation target and was measured again. When there was a disagreement, a unified conclusion was made through several discussions until a consensus was reached. The ICC ranged from 0 (no reliability) to 1 (perfect reliability)^29^. With repeated testing, the ICC met the criterion (> 0.80) in all the cases.

### Statistical methods

Data were analyzed using SPSS Statistics version 26.0 for Windows (IBM Corp., Armonk, NY, USA). Continuous variables are presented as mean and standard deviation (SD), and categorical variables are presented as frequency and percentage. The intra-rater reliability in muscle thickness and cross-sectional area measurements was assessed using the ICC coefficient, with a mean value of 0.83. Differences according to sex and group between Group 1 (TMJ arthralgia alone) and Group 2 (TMJ arthralgia with concurrent HATMD) were investigated using chi-square tests for categorical variables. One-way analysis of variance (ANOVA) with Tukey’s post-hoc test and Student’s t-tests were used for numeric variables. Chi-squared tests with Bonferroni-adjusted post hoc analyses were used to determine equality of proportions. Spearman's correlation coefficients (r) were calculated for VAS, demographics, and ultrasonographic parameters. The range is − 1 to + 1, with − 1 representing a perfect linear negative correlation and + 1 representing a perfect linear positive correlation. For all analyses, a two-tailed *p*-value of less than 0.05 was considered statistically significant.

### Institutional Review Board

The research protocol for this study was reviewed in compliance with the Helsinki Declaration and approved by the Institutional Review Board of Kyung Hee University Dental Hospital in Seoul, South Korea (KHD IRB, IRB No-KH-1709-4).

### Informed consent

Informed consent was obtained from all the subjects involved in the study.

## Results

### Demographics

Among 100 consecutive patients with TMJ arthralgia during the study period, Group 1 consisted of 86 patients with arthralgia alone (60 females and 26 males; mean age 41.15 ± 17.65 years), while Group 2 comprised 14 patients with concurrent arthralgia and HATMD (11 females and 3 males; mean age 33.00 ± 16.72 years). The difference in mean age between the groups was not significant (*p*-value = 0.111). The ratio of TMJ arthralgia alone versus co-occurrence of arthralgia and HATMD was 6.14:1. Analysis of the results revealed the TMD predominantly affected the females with a female-to-male ratio of 2.45:1. By group, the female-to-male ratio in Group 2 (2.31:1) was higher than Group 1 (3.67:1), but the difference was not statistically significant (*p*-value = 0.752). The ratio of patients in the acute phase to in the chronic phase was 1:3, and the mean symptom duration was 547.05 ± 955.64 days (18.23 ± 31.83 months). There were no significant differences in the distribution of sex, age, pain duration, and chronicity between the two groups. The pain area value was significantly higher in Group 2 than in Group 1 (2.23 ± 1.75 vs. 5.79 ± 2.39, *p*-value < 0.001). In addition, the mean VAS score was significantly higher in Group 2 than in Group 1 (3.41 ± 1.82 vs. 5.57 ± 12.14, *p*-value = 0.002). There was a significant difference in the frequency of pain between groups. In Group 1, pain was lateralized to the left side (52.3% vs. 28.6%) more frequently, and in Group 2, bilateral pain (11.6% vs. 50%) was observed more frequently (*p*-value = 0002) (Table 1).

**Table 1:** Demographics and pain characteristics of TMD patients.

	Total (n = 100)	Group 1 (Arthralgia)	Group 2 (Arthralgia with HATMD)	*p*-value
		Mean ± SD or n (%) (n = 86)	Mean ± SD or n (%) (n = 14)	
Demographics				
Age^a^	40.01 ± 17.67	41.15 ± 17.65	33.00 ± 16.72	0.111
Female sex^b^	71 (71.0)	60 (69.8)	11 (78.6)	0.752
Pain characteristics				
Pain duration (days)^a^	547.05 ± 955.64	513.78 ± 965.86	751.43 ± 895.98	0.375
Chronicity^c^				
(1) Acute phase (≤ 6 months)	25 (25.0)	24 (27.9)	1 (7.1)	0.179
(2) Chronic phase (> 6 months)	75 (75.0)	62 (72.1)	13 (92.9)	
Pain area^a^	2.73 ± 2.22	2.23 ± 1.75	5.79 ± 2.39	< 0.001***
VAS^a^	3.71 ± 2.01	3.41 ± 1.82	5.57 ± 12.14	0.002**
Pain laterality^c^				
Right side	34 (34.0)	31 (36.0)	3 (21.4)	0.002**
Left side	49 (49.0)	**45 (52.3)**	4 (28.6)	
Both sides	17 (17.0)	10 (11.6)	**7 (50.0)**	

*HATMD* headache attributed to temporomandibular disorder, *SD* standard deviation.

^a^Results were obtained via t- test.

^b^The results were obtained via a chi-square test (two-sided).

^c^Fisher’s exact test. A *p*-value < 0.05 was considered significant.

**: *p*-value < 0.01, ***: *p*-value < 0.001.

Significant results are in bold text.

### Thickness and cross-sectional area of masticatory muscle and gender-based differences

**Figure 3:**
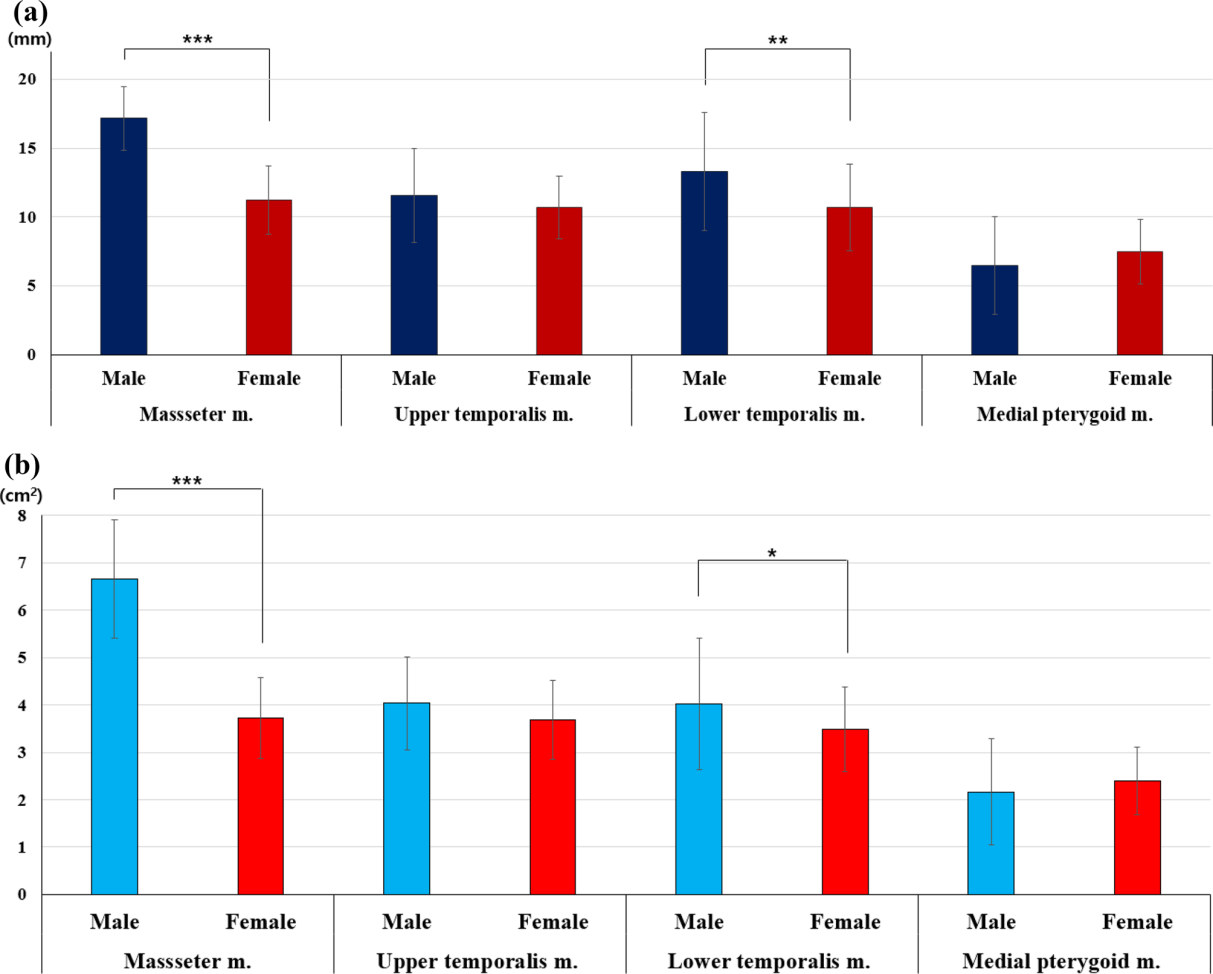
Sex-based differences in muscle thickness and cross-sectional area. (**a**) Thickness and (**b**) cross-sectional area.

On analysis, the thickest muscle was the masseter (12.58 ± 4.24 mm), followed by the upper temporalis (10.84 ± 5.04 mm) and lower temporalis muscles (11.19 ± 3.50 mm), and the thinnest muscle was the medial pterygoid (6.95 ± 2.24 mm) (*p*-value = 0.001). In terms of the cross-sectional area, the masseter muscle (4.46 ± 2.57 cm^2^) was the largest, followed by the upper temporalis (3.74 ± 1.93 cm^2^), lower temporalis (3.68 ± 0.98 cm^2^), and medial pterygoid muscles (2.33 ± 0.79 cm^2^) (*p*-value < 0.001) (Table 2). Muscles with differences between men and women were the masseter and lower temporalis muscles. Both thickness and cross-sectional area were more significant in males than in females (all *p*-value < 0.05) (Fig. 3).

**Table 2:** Comparison of thickness and cross-sectional area of the masticatory muscles and investigation of gender differences.

	Total (n = 100)	*p*-value^a^ (comparison according to masticatory muscles)	Post-hoc	Male (n = 29)	Female (n = 71)	*p*-value^b^ (comparison according to sex)
Thickness (mm)						
Masseter m.	12.58 ± 4.24	< 0.001***	Masseter m. > upper temporalis and medial pterygoid m	17.16 ± 2.33	11.21 ± 2.47	< 0.001***
Upper temporalis m.	10.84 ± 5.04		Upper temporalis m. > medial pterygoid m	11.58 ± 3.42	10.69 ± 2.29	0.135
Lower temporalis m.	11.19 ± 3.50		Lower temporalis m. > medial pterygoid m	13.31 ± 4.28	10.69 ± 3.15	0.001**
Medial pterygoid m.	6.95 ± 2.24		Medial pterygoid m < masseter, upper temporalis, and lower temporalis m	6.47 ± 3.53	7.46 ± 2.33	0.102
Cross-sectional area (cm^2^)						
Masseter m.	4.46 ± 2.57	< 0.001***	Masseter m. > upper temporalis, lower temporalis, and medial pterygoid m	6.66 ± 1.24	3.73 ± 0.85	0.001**
Upper temporalis m.	3.74 ± 1.93		Upper temporal m. > lower temporalis m	4.04 ± 0.98	3.68 ± 0.84	0.069
Lower temporalis m.	3.68 ± 0.98		Lower temporalis m. > medial pterygoid m	4.03 ± 1.38	3.49 ± 0.89	0.025*
Medial pterygoid m.	2.33 ± 0.79		Medial pterygoid m < masseter, upper temporalis, and lower temporalis m	2.17 ± 1.12	2.41 ± 0.71	0.202

The results are shown as mean ± SD.

*SD* standard deviation.

^a^the results were obtained via ANOVA and Tukey’s post-hoc test.

^b^Results were obtained via the Student’s t-test.

Statistical significance was set at *p*-value < 0.05. *: *p*-value < 0.05, **: *p*-value < 0.01, ***: *p*-value < 0.001.

The significant results are shown in bold.

### Comparison of masticatory muscle thickness and area between TMD groups

The differences between the TMD groups according to the absence or presence of HATMD were examined (Table 3).

**Table 3:** Comparison of the masticatory muscle thickness and cross-sectional area between the TMD groups.

	Group 1 (Arthralgia)	Group 2 (Arthralgia with HATMD)	*p*-value
	Mean ± SD (n = 86)	Mean ± SD (n = 14)	
Thickness (mm)			
Masseter m.			
Rt.	13.42 ± 3.33	9.96 ± 4.14	0.001**
Lt.	12.05 ± 3.54	13.23 ± 16.01	0.545
Average	12.74 ± 3.05	11.59 ± 8.64	0.352
Upper temporalis m.			
Rt.	11.13 ± 2.63	9.88 ± 2.79	0.137
Lt.	9.78 ± 2.92	10.24 ± 3.01	0.601
Average	10.96 ± 5.34	10.06 ± 2.58	0.537
Lower temporalis m.			
Rt.	11.61 ± 3.64	10.48 ± 3.99	0.293
Lt.	11.25 ± 4.43	9.06 ± 3.41	0.045*
Average	11.43 ± 3.46	9.77 ± 3.56	0.101
Medial pterygoid m.			
Rt.	7.21 ± 2.83	6.99 ± 2.30	0.759
Lt.	6.56 ± 2.21	7.74 ± 2.93	0.081
Average	6.89 ± 2.27	7.37 ± 2.09	0.460
Cross-sectional area (cm^2^)			
Masseter m.			
Rt.	4.81 ± 4.41	3.16 ± 0.98	0.003**
Lt.	4.53 ± 3.02	3.20 ± 1.00	0.002**
Average	4.67 ± 2.69	3.18 ± 0.92	0.004**
Upper temporalis m.			
Rt.	3.82 ± 0.91	3.57 ± 0.73	0.254
Lt.	3.75 ± 3.82	3.32 ± 0.86	0.362
Average	3.79 ± 2.06	3.45 ± 0.76	0.539
Lower temporalis m.			
Rt.	3.70 ± 1.06	3.31 ± 1.17	0.250
Lt.	3.81 ± 1.16	3.11 ± 0.97	0.025*
Average	3.76 ± 0.95	3.21 ± 1.02	0.049*
Medial pterygoid m.			
Rt.	2.35 ± 0.88	2.27 ± 0.71	0.727
Lt.	2.28 ± 0.99	2.60 ± 0.92	0.243
Average	2.31 ± 0.81	2.44 ± 0.71	0.593

Group 1: arthralgia alone group, Group 2: arthralgia with headache attributed to temporomandibular disorder group, SD, standard deviation; The results were obtained via Student’s t-test.

Statistical significance was set at *p*-value < 0.05. *: *p*-value < 0.05, **: *p*-value < 0.01.

The significant results are shown in bold.

**Figure 4:**
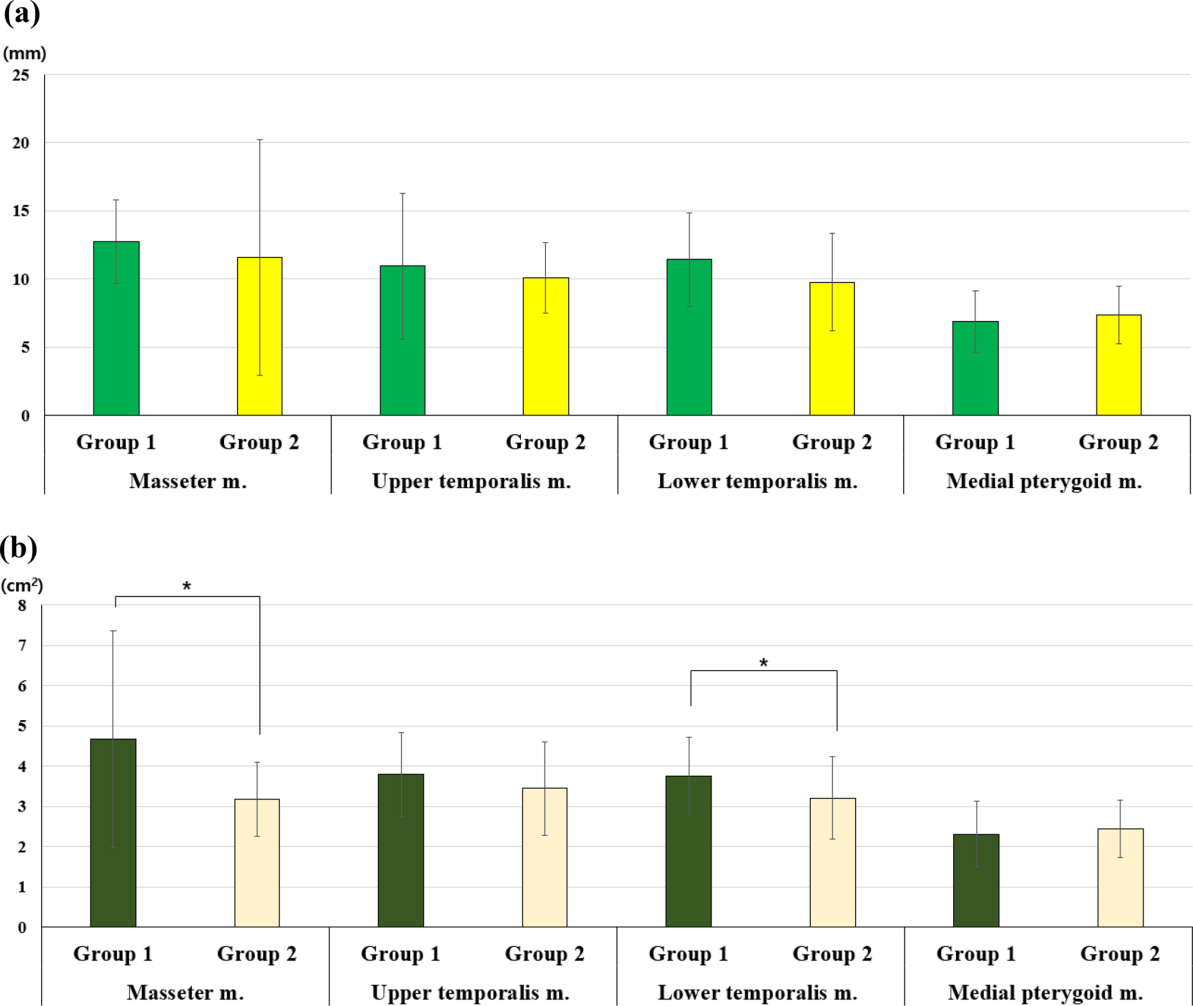
Differences in muscle thickness and cross-sectional area among the TMD groups. (**a**) Thickness and (**b**) cross-sectional area.

For muscle thickness, the right masseter muscle (13.42 ± 3.33 mm vs. 9.96 ± 4.14 mm, *p*-value = 0.001) and left lower temporalis muscle (11.25 ± 4.43 mm vs. 9.06 ± 3.41 mm, *p*-value = 0.045) were significantly thinner in Group 2 than in Group 1. The cross-sectional area of the masseter (4.67 ± 2.69 cm^2^ vs. 3.18 ± 0.92 cm^2^, *p* = 0.04) and the lower temporalis muscles (3.76 ± 0.95 cm^2^ vs. 3.21 ± 1.02 cm^2^, *p* = 0.049) was significantly smaller in Group 2 than in Group 1 (Fig. 4).

### Implications of pain laterality

In the analysis between the TMD groups, additional analysis was performed according to the pain laterality (Table 4). In Group 1, the upper temporalis muscle thickness was significantly less in patients with pain on the right side than in patients with bilateral pain (3.53 ± 0.83 cm^2^ vs. 4.30 ± 0.91 cm^2^, *p*-value = 0.040). However, except for this case, there was no significant difference in the thickness and cross-sectional area of the masticatory muscle according to the pain laterality (right or left) and the factors that occurred on one or both sides. That is, the existence of TMD pain in either the right, left, or both sides did not cause a statistically significant difference in both the cross-sectional area and muscle thickness of the masseter, lower temporalis, and medial pterygoid muscles among the masticatory muscles.

**Table 4:** Comparison of muscle thickness and cross-sectional area values according to the pain laterality.

	Side	Group 1 (Arthralgia) (n = 86)				F-value of group 1	Group 2 (Arthralgia with HATMD) (n = 14)				F-value of group 2
		Rt. pain (n = 31) mean ± SD	Lt. pain (n = 45) mean ± SD	Pain on both sides (n = 10) mean ± SD	*p*-value		Rt. pain (n = 3) mean ± SD	Lt. pain (n = 4) mean ± SD	Pain on both sides (n = 7) mean ± SD	*p*-value	
Muscle thickness (mm)											
Masseter m	Rt.	13.54 ± 3.33	13.07 ± 3.18	14.65 ± 3.94	0.389	0.955	7.28 ± 1.49	11.60 ± 7.16	10.17 ± 2.32	0.419	0.943
	Lt.	12.09 ± 3.50	12.25 ± 3.35	11.06 ± 4.64	0.632	0.461	8.15 ± 1.43	11.30 ± 3.63	16.50 ± 22.81	0.753	0.291
Upper temporalis m.	Rt.	10.45 ± 2.17	11.32 ± 2.78	12.35 ± 2.93	0.107	2.295	9.15 ± 1.84	9.28 ± 3.88	10.55 ± 2.68	0.707	0.357
	Lt.	9.49 ± 1.93	10.28 ± 3.19	17.22 ± 28.29	0.084	1.965	9.14 ± 0.56	11.98 ± 3.35	9.72 ± 3.32	0.412	0.963
Lower temporalis m.	Rt.	11.67 ± 3.69	11.48 ± 3.76	11.98 ± 3.20	0.922	0.081	9.23 ± 1.12	11.93 ± 5.55	10.20 ± 4.04	0.687	0.388
	Lt.	11.14 ± 3.65	11.11 ± 4.30	12.16 ± 7.03	0.789	0.238	7.94 ± 1.28	9.58 ± 3.88	9.24 ± 4.04	0.830	0.190
Medial pterygoid m.	Rt.	7.36 ± 2.98	6.86 ± 2.69	8.31 ± 2.91	0.318	1.162	7.47 ± 3.29	7.53 ± 2.71	6.48 ± 1.89	0.742	0.307
	Lt.	6.53 ± 2.23	6.47 ± 2.30	7.11 ± 1.79	0.711	0.342	7.57 ± 3.25	6.60 ± 1.43	8.47 ± 3.54	0.630	0.482
Cross-sectional area (cm^2^)											
Masseter m.	Rt.	5.72 ± 7.17	4.22 ± 1.07	4.62 ± 1.32	0.347	1.072	2.53 ± 0.42	3.33 ± 1.68	3.34 ± 0.60	0.485	0.772
	Lt.	4.05 ± 1.15	4.85 ± 4.02	4.57 ± 1.18	0.527	0.646	2.98 ± 0.46	3.83 ± 1.44	2.93 ± 0.83	0.360	1.122
Upper temporalis m.	Rt.	3.53 ± 0.83	3.92 ± 0.93	4.30 ± 0.91	0.040*	3.351	3.14 ± 0.32	3.39 ± 0.78	3.85 ± 0.78	0.338	1.201
	Lt.	3.25 ± 0.63	4.13 ± 5.23	3.63 ± 0.96	0.611	0.496	3.17 ± 0.39	3.34 ± 0.70	3.38 ± 1.14	0.949	0.053
Lower temporalis m.	Rt.	3.63 ± 1.19	3.68 ± 1.00	4.03 ± 0.96	0.587	0.536	3.34 ± 0.56	3.58 ± 1.55	3.14 ± 1.25	0.854	0.160
	Lt.	3.56 ± 1.03	3.98 ± 1.20	3.82 ± 1.39	0.321	1.151	2.87 ± 0.11	3.11 ± 0.86	3.21 ± 1.28	0.895	0.112
Medial pterygoid m.	Rt.	2.45 ± 1.12	2.24 ± 0.70	2.51 ± 0.75	0.499	0.702	2.64 ± 1.06	2.10 ± 0.29	2.21 ± 0.77	0.620	0.500
	Lt.	2.15 ± 0.73	2.36 ± 1.21	2.31 ± 0.49	0.674	0.396	2.57 ± 1.14	2.36 ± 0.28	2.76 ± 1.13	0.813	0.211

Group 1: arthralgia alone; Group 2: arthralgia with headache attributed to temporomandibular disorder. The results were obtained using ANOVA and Tukey’s post-hoc tests.

Statistical significance was set at *p*-value < 0.05. *: *p*-value < 0.05.

The significant results are shown in bold.

### Correlation coefficient (r) between VAS and masticatory muscle changes

The increase in VAS score was significantly negatively correlated with the thickness of the masseter (r = − 0.268, *p*-value < 0.01) and lower temporalis muscles (r = − 0.215, *p*-value < 0.05). Furthermore, the increase in VAS was correlated with the cross-sectional area of the masseter (r = − 0.329, *p*-value < 0.01) and lower temporalis muscles (r = − 0.293, *p*-value < 0.05) (Table 5).

**Table 5:** Evaluation of correlation coefficient (r) between VAS and masticatory muscle changes.

Correlation coefficient (r) (n = 100)	Female sex	Age	Headache attributed to TMD	Pain area	Pain duration	Masseter m. thickness	Upper temporalis m. thickness	Lower temporalis m. thickness	Medial pterygoid m. thickness	Masseter m area	Upper temporalis m area	Lower temporalis m area	Medial pterygoid m area
VAS	0.140	0.093	0.362**	0.462**	− 0.043	− 0.268**	− 0.155	− 0.215*	0.057	− 0.329**	− 0.109	− 0.293**	0.066
Female sex		0.204*	0.067	0.144	0.015	− 0.684**	− 0.091	− 0.266**	0.333**	− 0.606**	− 0.166	− 0.246*	0.317**
Age			− 0.166	− 0.034	− 0.293**	− 0.134	0.088	0.028	− 0.087	− 0.062	− 0.035	− 0.074	− 0.135
Headache attributed to TMD				0.489**	0.198*	− 0.277**	− 0.101	− 0.224*	0.088	− 0.370**	− 0.093	− 0.168	0.083
Pain area					0.123	− 0.184	− 0.081	− 0.303**	0.017	− 0.202*	0.001	− 0.196	0.012
Pain duration						− 0.066	− 0.001	− 0.024	0.015	− 0.059	− 0.017	0.015	0.028
Masseter m. thickness							0.193	0.421**	− 0.247*	0.826**	0.229*	0.522**	− 0.206*
Upper temporalis m. thickness								0.213*	0.020	0.157	0.617**	0.093	0.006
Lower temporalis m. thickness									0.164	0.439**	0.038	0.701**	0.113
Medial pterygoid m. thickness										− 0.271**	**− **0.107	0.083	0.904**
Masseter m. area											0.204*	0.557**	− 0.227*
Upper temporalis m. area												0.080	**− **0.035
Lower temporalis m. area													0.161

The results were obtained using Spearman’s correlation analysis.

Statistical significance was set at *p*-value < 0.05. *: *p*-value < 0.05, **: *p*-value < 0.01.

The significant results are shown in bold.

In other words, the results indicate that pain intensity significantly increases as the thickness of the masseter and lateral muscles decreases in patients with TMJ arthralgia.

The thickness and cross-sectional areas of the masseter (r = 0.826), upper temporalis (r = 0.617), and lower temporalis muscles (r = 0.701) were strongly correlated (all *p*-value < 0.01). However, this correlation was not observed for the medial pterygoid muscle. When examining the correlation between the muscles, masseter muscle thickness showed a significant positive correlation with that of the lower temporalis muscle (r = 0.421, *p*-value < 0.05). The thickness of the upper and lower temporalis muscles also showed a significant positive correlation (r = 0.213, *p*-value < 0.05).

## Discussion

In the present study, the thickness and cross-sectional area of the four major masticatory muscles were investigated using ultrasonography in patients with TMJ arthralgia. Thickness and cross-sectional area were higher in the masseter muscle than in the upper temporalis, external pterygoid, and medial pterygoid muscles. The measures of the masseter and lower temporalis muscles were significantly greater in males than in females. As hypothesized, patients with TMJ arthralgia and HATMD had a more significant reduction in the cross-sectional area of the masseter and lower temporalis muscles than in those with TMJ arthralgia alone. HATMD is a relatively newly recognized type of headache^15,30^, and its characterization is somewhat ambiguous. HATMD can co-occur with primary headaches such as tension-type headaches or migraines^31^. Indeed, there are numerous overlapping symptoms between primary headaches and TMD^32,33^. However, clearly elucidating their connection still poses a challenge. Moreover, these muscle thickness and area reductions were associated with increased pain intensity. In patients with arthralgia and HATMD, the VAS score, a scale of subjective pain intensity, was significantly higher than those with arthralgia alone. The TMJ arthralgia with HATMD group had a higher proportion of females compared to the TMJ arthralgia alone group (78.6% vs. 69.8%, *p*-value = 0.752). In previous pain studies, it has been observed that women tend to experience longer durations, wider-ranging symptoms, and more severe and diverse manifestations compared to men^34,35^. Although studies examining the female-to-male ratio of HATMD are very limited, it's noteworthy that headaches, such as tension-type headaches and migraines, are more prevalent in women than in men^36,37^. Therefore, the female-to-male ratio becomes a point of caution when interpreting the results of our study. Spearman’s correlation analysis revealed that the increase in VAS correlated negatively with the thickness and cross-sectional area of the masseter and lower temporalis muscles. This study is the first to examine the major masticatory muscles of patients with TMD using ultrasonography. It is noteworthy that the study revealed the clinical and imaging characteristics according to the presence or absence of HATMD.

Interestingly, sex-based differences in thickness and cross-sectional area were observed only in the masseter and lower temporalis muscles but not in the upper temporalis and medial pterygoid muscles. In a previous study examining sex-based differences in skeletal muscle, there were differences in the number of muscle fibers and the distribution of type I and II fibers in some, but not all, muscles^38^. In this study, the masseter thickness in the relaxed state was 17.16 ± 2.33 mm in males and 11.21 ± 2.47 mm in females. The masseter muscle exerts the greatest masticatory force among the four major masticatory muscles and is also the muscle with which TMD pain occurs most commonly^39,40^. Satiroğlu et al. reported similar values for thickness in the relaxed state, and the thickness of the masseter in males was 15.5 ± 2.0 mm, and in females was 12.1 ± 1.9 mm, respectively^41^. In healthy Korean adults, the masseter muscle thickness measured by ultrasonography was 9.8 ± 1.3 mm in females and 11.3 ± 1.2 mm in males^42^. These differences were probably because the reference points for measurement, age, and TMD disease status differed. Although this value is lower than those found in our study, it is consistent with the fact that the masseter muscle thickness of males is greater than that of females. Because the masseter muscle is located in the superficial layer, it is relatively easier to measure with ultrasound than other masticatory muscles. However, in this context, ultrasonography is not widely used to measure masticatory muscles^43^. In particular, reliable ultrasonographic measurements are difficult because the lower temporalis, lateral pterygoid, and medial pterygoid muscles are located deep to the masseter muscle.

Decreased masseter muscle volume is associated with increased pain intensity and pain area. Skeletal muscle atrophy causes pain, weakness, and discomfort owing to muscle fiber thinning^44^. Muscle loss indicates the onset of many local conditions, such as muscular dystrophy, cancer, and nerve damage^45^. Puthucheary et al. reported that muscle layer thickness and cross-sectional area were comparative markers of muscle wasting and weakness in the rectus femoris muscle^46^. Conversely, if bruxism persists, masseter hypertrophy may occur^47^, and masseter muscle volume can increase. Bruxism is characterized by episodes of heightened muscle activity involving clenching or rhythmic contractions of the masticatory muscles^48^. However, repetitive clenching and grinding movements, along with functional hyperactivity due to bruxism, tend to induce traumatic injuries to the masticatory system^49^. In addition, sleep bruxism, often associated with microarousals during sleep, can cause issues in the masticatory muscles. Further ultrasound-based studies will be necessary to elucidate the relationship between bruxism, orofacial pain, and muscle weakness or hypertrophy.

Additional studies with long-term follow-up on 2D and 3D anatomical changes and muscle pain intensity in patients with TMD are required. In this study, the thickness of each masticatory muscle and its cross-sectional area showed a very strong positive correlation. Muscle thickness measured using ultrasonography correlates with the cross-sectional area measured using CT and MRI at one-time point^50^. Patients with a spectrum of neuromuscular disorders have muscle thickness that correlates strongly with disability score and muscle strength^51^. Conversely, when vastus lateralis muscle thickness was increased through physical training in patients with chronic low back pain, pain intensity decreased^50^. These findings suggest that the decrease in volume of the masseter and lower temporalis muscles in patients with TMD is related to the increase in pain, but care should be taken in interpreting the limited data.

In the examination of the temporalis muscle, there was no difference between the sexes or TMD groups in the upper part of the temporal fascia; however, the lower part attached to the coronoid process differed. The temporalis muscle functions as the main retractor of the mandible during mastication^52^. Contraction of the posterior fibers of the temporalis muscle results in retrusion of the mandible, and contraction of the anterior fibers moves the mandible in a dorso-cranial direction, elevating the mandible^53^. Measuring muscle thickness using ultrasonography is a good surrogate for measuring muscle mass and has excellent intra- and inter-observer reliability^54,55^. Decreased muscle thickness reflects muscle weakness^56^. In this study, the temporalis muscle was divided into upper and lower parts that were clearly distinguished in ultrasonographic examination. As previous studies examining changes in the upper and lower parts of the temporalis muscles are limited, further investigation is needed to understand why muscle changes or specific relationships were only present in the lower temporalis muscles. The increase in pain intensity was negatively correlated with the thickness and cross-sectional area of the lower temporalis muscles. In addition, the decrease in the cross-sectional area of the masseter and lower temporalis muscles in patients with TMJ arthralgia and HATMD was more pronounced than that in patients with TMJ arthralgia alone.

Hypotrophy and masticatory muscle contracture may be associated with TMJ sounds, TMD pain, and headaches in patients with TMD^57^. In previous electromyogram results, the muscle fatigue of the masseter and temporalis muscles correlated with the increased intensity of the temporomandibular dysfunction index in TMD patients^58^. Thus, decreased anatomical and weakened pathological masseter and lower temporalis musculature can reflect pain and HATMD in patients with arthralgia. The temporalis muscle is anatomically divided into anterior, middle, and posterior parts^53^. However, these three regions are not clearly distinguished in ultrasonography examination. This study found out which HATMD is associated with either the upper or lower part of the temporalis muscle. The upper temporalis muscle, similar to the masseter muscle, is a superficial masticatory muscle; therefore, it can be easily and reliably measured using ultrasonography. The upper temporalis muscle thickness has been measured previously, and decreased upper temporalis muscle thickness has been reported to be associated with disease progression and reduced survival rates^59,60^. The thickness of the upper temporalis muscle was 11.58 ± 3.42 mm in males and 10.69 ± 2.29 mm in females, respectively. There was no significant difference in upper temporalis muscle thickness and cross-sectional area according to sex or TMD group. According to the DC/TMD for HATMD, pain in the upper temporalis area related to the function and parafunction of the TMJ and pain in the provocation test should be present in the upper temporalis muscle^30^. Electromyography showed that the activity of the masseter and upper temporalis muscles increased in patients with TMD compared to the controls^61^. Chronic stress increases the electrical activity of the upper temporalis muscles in TMD^62^. Therefore, it was hypothesized that thickness and cross-sectional area changes in the upper temporalis muscle would be more prominent in patients with HATMD than in those with TMJ arthralgia alone, but this hypothesis has not been proven. Further investigations are needed to clarify the relationship between anatomical changes in the upper temporalis muscle and TMJ arthralgia or comorbid HATMD.

This study had several limitations. Since this was a cross-sectional study without a regular control group, only comparisons of masticatory muscles between sexes and TMD groups were performed. In addition, we investigated the effect of HATMD comorbidity on TMJ arthralgia in 100 patients; however, the number of patients with HATMD was limited to 14. Although not statistically significant, the female-to-male ratio was higher in the TMJ arthralgia with HATMD group than in the TMJ arthralgia alone group. This sex ratio discrepancy may cause bias in measurements of muscle thickness and cross-sectional area. Conversely, it can be interpreted that HATMD is more likely to co-occur with arthralgia in women compared to men. This study is significant in that it investigated all four major masticatory muscles using ultrasonography for the first time; however, there are clear limitations in fundamental ultrasonographic evaluation. Among the four main masticatory muscles, the lateral pterygoid muscle is located deep to the masseter and lower temporalis muscles and was not investigated because of the difficulty in obtaining accurate measurements using the ultrasound device. Because the waves used in ultrasonography show continuously changing patterns, it takes considerable experience to perform ultrasonic measurements. A single observer with more than 10 years of experience in the field of orofacial pain evaluated the muscles, but there is a possibility of observer bias. In addition, the force applied to the tissue and each patient's unique anatomy can result in different images. Therefore, ultrasound evaluation can be more subjective than CBCT, CT, and MRI, which can produce clearer images. Consequently, ultrasonography is not commonly used. Therefore, efforts to accumulate experience and research with imaging modalities such as CT and MRI that have precise results will be continuously needed.

## Conclusions

This study is the first to examine the major masticatory muscles of patients with TMD using ultrasonography. The clinical and imaging characteristics according to the presence or absence of HATMD were also clarified. Patients with TMJ arthralgia and HATMD had a more significant reduction in the cross-sectional area of the masseter and lower temporalis muscles than those with TMJ arthralgia alone. Our findings can be used with high value for the evaluation and treatment of masticatory muscles in patients with TMJ arthralgia. To clarify our findings, further longitudinal studies tracking changes in the cross-sectional area and thickness of the masticatory muscles over time are needed.

## Data Availability

The datasets used and/or analyzed during the current study are available from the corresponding author upon reasonable request.

## References

[1] Schiffman E (2014). Diagnostic criteria for temporomandibular disorders (DC/TMD) for clinical and research applications: recommendations of the International RDC/TMD Consortium Network* and Orofacial Pain Special Interest Group†. J Oral Facial Pain Headache.

[2] Dworkin SF (2010). Research diagnostic criteria for temporomandibular disorders: Current status & future relevance. J. Oral Rehabil..

[3] Headache Classification Committee of the International Headache Society (IHS) The International Classification of Headache Disorders, 3rd edition. *Cephalalgia* **38**, 1–211. 10.1177/0333102417738202 (2018). 10.1177/0333102417738202 29368949

[4] Valesan LF (2021). Prevalence of temporomandibular joint disorders: a systematic review and meta-analysis. Clin. Oral Investig..

[5] Bagis B, Ayaz EA, Turgut S, Durkan R, Özcan M (2012). Gender difference in prevalence of signs and symptoms of temporomandibular joint disorders: A retrospective study on 243 consecutive patients. Int. J. Med. Sci..

[6] Ballegaard V, Thede-Schmidt-Hansen P, Svensson P, Jensen R (2008). Are headache and temporomandibular disorders related? A blinded study. Cephalalgia.

[7] van der Meer HA (2017). The association between headaches and temporomandibular disorders is confounded by bruxism and somatic symptoms. Clin. J. Pain.

[8] Cooper BC, Kleinberg I (2007). Examination of a large patient population for the presence of symptoms and signs of temporomandibular disorders. Cranio.

[9] Kothari SF (2016). Pain profiling of patients with temporomandibular joint arthralgia and osteoarthritis diagnosed with different imaging techniques. J. Headache Pain.

[10] Chen D (2017). Osteoarthritis: Toward a comprehensive understanding of pathological mechanism. Bone Res..

[11] Guo Q (2018). Rheumatoid arthritis: Pathological mechanisms and modern pharmacologic therapies. Bone Res..

[12] Vivaldi D (2018). Headache attributed to TMD Is associated with the presence of comorbid bodily pain: A case-control study. Headache.

[13] Effat KG (2021). A comparative clinical study of arthrogenous versus myogenous temporomandibular disorder in patients presenting with Costen's syndrome. Cranio.

[14] Naeije M, Hansson TL (1986). Electromyographic screening of myogenous and arthrogenous TMJ dysfunction patients. J. Oral Rehabil..

[15] Tchivileva IE (2021). Clinical, psychological, and sensory characteristics associated with headache attributed to temporomandibular disorder in people with chronic myogenous temporomandibular disorder and primary headaches. J. Headache Pain.

[16] Costa YM (2015). Headache attributed to masticatory myofascial pain: clinical features and management outcomes. J. Oral Facial Pain Headache.

[17] Costa YM (2016). Headache attributed to masticatory myofascial pain: Impact on facial pain and pressure pain threshold. J. Oral Rehabil..

[18] Svensson P (2007). Muscle pain in the head: overlap between temporomandibular disorders and tension-type headaches. Curr. Opin. Neurol..

[19] Ekberg E, Vallon D, Nilner M (2002). Treatment outcome of headache after occlusal appliance therapy in a randomised controlled trial among patients with temporomandibular disorders of mainly arthrogenous origin. Swed. Dent. J..

[20] Carovac A, Smajlovic F, Junuzovic D (2011). Application of ultrasound in medicine. Acta Inform. Med..

[21] Stoustrup P (2017). Clinical orofacial examination in juvenile idiopathic arthritis: International consensus-based recommendations for monitoring patients in clinical practice and research studies. J. Rheumatol..

[22] Lee YH, Won JH, Kim S, Auh QS, Noh YK (2022). Advantages of deep learning with convolutional neural network in detecting disc displacement of the temporomandibular joint in magnetic resonance imaging. Sci. Rep..

[23] Pillen S, van Alfen N (2011). Skeletal muscle ultrasound. Neurol. Res..

[24] Chang P-H, Chen Y-J, Chang K-V, Wu W-T, Özçakar L (2020). Ultrasound measurements of superficial and deep masticatory muscles in various postures: reliability and influencers. Sci. Rep..

[25] Serra MD, Duarte Gavião MB, dos Santos Uchôa MN (2008). The use of ultrasound in the investigation of the muscles of mastication. Ultrasound Med. Biol..

[26] Lee Y-H, Auh QS (2022). Comparison of sleep quality deterioration by subgroup of painful temporomandibular disorder based on diagnostic criteria for temporomandibular disorders. Sci. Rep..

[27] Chen YJ, Chang PH, Chang KV, Wu WT, Özçakar L (2018). Ultrasound guided injection for medial and lateral pterygoid muscles: A novel treatment for orofacial pain. Med. Ultrason.

[28] Bianchi S (2020). Ultrasound and bone: A pictorial review. J. Ultrasound.

[29] Koo TK, Li MY (2016). A guideline of selecting and reporting intraclass correlation coefficients for reliability research. J. Chiropr. Med..

[30] Hara K (2016). Headache attributed to temporomandibular disorders and masticatory myofascial pain. J. Oral Sci..

[31] Ferrillo M (2023). Temporomandibular disorders and neck pain in primary headache patients: A retrospective machine learning study. Acta Odontol. Scand..

[32] Schiller J (2024). Effects on temporomandibular disorder in the treatment of tension-type headache with acupuncture and therapeutic exercises. A secondary analysis from a randomized controlled trial. Clin. Rehabil..

[33] Cruz D (2022). Genetic overlap between temporomandibular disorders and primary headaches: A systematic review. Jpn. Dent. Sci. Rev..

[34] Casale R (2021). Pain in women: A perspective review on a relevant clinical issue that deserves prioritization. Pain Ther..

[35] Bartley EJ, Fillingim RB (2013). Sex differences in pain: A brief review of clinical and experimental findings. Br. J. Anaesth..

[36] Rossi MF (2022). Sex and gender differences in migraines: A narrative review. Neurol. Sci..

[37] Onan D (2023). Debate: differences and similarities between tension-type headache and migraine. J. Headache Pain.

[38] Miller AEJ, MacDougall JD, Tarnopolsky MA, Sale DG (1993). Gender differences in strength and muscle fiber characteristics. Eur. J. Appl. Physiol. Occup. Physiol..

[39] Pyo CY, Kim TH, Kim DH (2021). Association between masticatory muscle activity and oral conditions in young female college students. Anat. Cell Biol..

[40] Lee YH, Lee KM, Auh QS (2021). MRI-based assessment of masticatory muscle changes in TMD patients after whiplash injury. J. Clin. Med..

[41] Satiroğlu F, Arun T, Işik F (2005). Comparative data on facial morphology and muscle thickness using ultrasonography. Eur. J. Orthod..

[42] Park KM (2018). The relationship between masseter muscle thickness measured by ultrasonography and facial profile in young Korean adults. Imaging Sci. Dent..

[43] Tircoveluri S (2013). Correlation of masseter muscle thickness and intermolar width—An ultrasonography study. J. Int. Oral Health.

[44] Powers SK, Lynch GS, Murphy KT, Reid MB, Zijdewind I (2016). Disease-induced skeletal muscle atrophy and fatigue. Med. Sci. Sports Exerc..

[45] Langer HT (2018). Muscle atrophy due to nerve damage is accompanied by elevated myofibrillar protein synthesis rates. Front. Physiol..

[46] Puthucheary ZA (2017). Rectus femoris cross-sectional area and muscle layer thickness: comparative markers of muscle wasting and weakness. Am. J. Respir. Crit. Care Med..

[47] Singh S, Shivamurthy DM, Agrawal G, Varghese D (2011). Surgical management of masseteric hypertrophy and mandibular retrognathism. Natl. J. Maxillofac. Surg..

[48] Shetty S, Pitti V, Satish Babu CL, Surendra Kumar GP, Deepthi BC (2010). Bruxism: A literature review. J. Indian Prosthodont. Soc..

[49] Kirveskari P, Jämsä T (2009). Health risk from occlusal interferences in females. Eur. J. Orthod..

[50] Franchi MV (2018). Muscle thickness correlates to muscle cross-sectional area in the assessment of strength training-induced hypertrophy. Scand. J. Med. Sci. Sports.

[51] Abraham A (2019). Muscle thickness measured by ultrasound is reduced in neuromuscular disorders and correlates with clinical and electrophysiological findings. Muscle Nerve.

[52] Visser A, McCarroll RS, Naeije M (1992). Masticatory muscle activity in different jaw relations during submaximal clenching efforts. J. Dent. Res..

[53] Yu SK, Kim TH, Yang KY, Bae CJ, Kim HJ (2021). Morphology of the temporalis muscle focusing on the tendinous attachment onto the coronoid process. Anat. Cell Biol..

[54] Hadda V (2017). Intra- and inter-observer reliability of quadriceps muscle thickness measured with bedside ultrasonography by critical care physicians. Indian J. Crit. Care Med..

[55] Hadda V (2017). Inter- and intra-observer variability of ultrasonographic arm muscle thickness measurement by critical care physicians. J. Postgrad. Med..

[56] Hadda V (2018). Trends of loss of peripheral muscle thickness on ultrasonography and its relationship with outcomes among patients with sepsis. J. Intensive Care.

[57] D’lppolito SM, Borri Wolosker AM, D’lppolito G, Herbert de Souza B, Fenyo-Pereira M (2010). Evaluation of the lateral pterygoid muscle using magnetic resonance imaging. Dentomaxillofac. Radiol..

[58] Woźniak K, Lipski M, Lichota D, Szyszka-Sommerfeld L (2015). Muscle fatigue in the temporal and masseter muscles in patients with temporomandibular dysfunction. Biomed. Res. Int..

[59] Furtner J (2018). Temporal muscle thickness is an independent prognostic marker in melanoma patients with newly diagnosed brain metastases. J. Neurooncol..

[60] Lee B (2021). Temporalis muscle thickness as an indicator of sarcopenia predicts progression-free survival in head and neck squamous cell carcinoma. Sci. Rep..

[61] Lauriti L (2014). Influence of temporomandibular disorder on temporal and masseter muscles and occlusal contacts in adolescents: an electromyographic study. BMC Musculoskeletal. Disord..

[62] Schmitter M, Kares-Vrincianu A, Kares H, Malsch C, Schindler HJ (2019). Chronic stress and temporalis muscle activity in TMD patients and controls during sleep: A pilot study in females. Clin. Oral Investig..

